# Correction: Synthesis and characterization of novel post-chain extension flame retardant waterborne polyurethane

**DOI:** 10.1039/d5ra90071a

**Published:** 2025-06-06

**Authors:** Gang Wu, Jinqing Li, Chunpeng Chai, Zhen Ge, Jialun Lin, Yunjun Luo

**Affiliations:** a School of Materials Science and Engineering, Beijing Institute of Technology Beijing 100081 PR China yjluo@bit.edu.cn +86 0 10 68913698 +86 0 10 68913698

## Abstract

Correction for ‘Synthesis and characterization of novel post-chain extension flame retardant waterborne polyurethane’ by Gang Wu *et al.*, *RSC Adv.*, 2015, **5**, 97710–97719, DOI: 10.1039/C5RA12975C.

The Royal Society of Chemistry is publishing this notice to alert readers that the authors published a related paper at the same time in *Polymer Degradation and Stability*^1^ which should have been cited in this *RSC Advances* article. While there is new work reported in both articles, Fig. 3–5, 8, 10 and 11, and Tables 5–7 of the *RSC Advances* article are also published in ref. 1. There is also a significant amount of text overlap between both articles.

The authors have not responded to any correspondence regarding this matter nor the wording of this notice.

The Royal Society of Chemistry apologises for these errors and any consequent inconvenience to authors and readers.
